# The PGE_2 _EP2 receptor and its selective activation are beneficial against ischemic stroke

**DOI:** 10.1186/2040-7378-2-12

**Published:** 2010-07-08

**Authors:** Muzamil Ahmad, Sofiyan Saleem, Zahoor Shah, Takayuki Maruyama, Shuh Narumiya, Sylvain Doré

**Affiliations:** 1 Department of Anesthesiology and Critical Care Medicine, Johns Hopkins University, Baltimore, Maryland, 21205, USA; 2 Pharmacological Research Laboratories, Ono Pharmaceutical Co. Ltd., Mishima-gun, Osaka, Japan; 3 Department of Pharmacology, Kyoto University Faculty of Medicine, Kyoto 606-8501, Japan; 4 Department of Pharmacology and Molecular Sciences, Johns Hopkins University, Baltimore, Maryland, 21205, USA

## Abstract

**Background:**

The prostaglandin E_2 _EP2 receptor has been shown to be important in dictating outcomes in various neuroinflammatory disorders. Here, we investigated the importance of the EP2 receptor in short- and long-term ischemic outcomes by subjecting wildtype (WT) and EP2 knockout (EP2^-/-^) mice to two distinct and complementary stroke models [transient and permanent middle cerebral artery occlusion (tMCAO and pMCAO)] and by using the EP2 receptor agonist ONO-AE1-259-01.

**Methods:**

First, WT and EP2^-/- ^mice were subjected to 90-min tMCAO with a monofilament followed by 4-day reperfusion. Second, WT mice were infused intracerebroventricularly with vehicle or ONO-AE1-259-01 45-50 min before being subjected to tMCAO. Finally, WT and EP2^-/- ^mice were subjected to pMCAO and allowed to survive for an extended period of 7 days.

**Results:**

Infarct volumes in EP2^-/- ^mice were 55.0 ± 9.1% larger after tMCAO and 33.3 ± 8.6% larger after pMCAO than those in WT mice. Neurobehavioral deficits also were significantly greater in the EP2^-/- ^mice. These results suggest that EP2 is beneficial and that activation is sustained for days after the stroke. We also found that pharmacologic activation of EP2 with 1.0- and 2.0-nmol doses of ONO-AE1-259-01 was sufficient to significantly reduce the infarct volume in WT mice compared with that in vehicle-treated controls (20.1 ± 3.9% vs. 37.1 ± 4.6%). This reduction correlated with improved neurologic scores. No significant effect on physiologic parameters was observed.

**Conclusion:**

Together, our results reveal that pharmacologic stimulation of the EP2 receptor has an important beneficial role in cerebral ischemia and might be considered as an adjunct therapy for ischemic stroke.

## Introduction

Prostaglandin E_2 _(PGE_2_), formed from arachidonic acid by the action of cyclooxygenases and PGE_2 _synthases, was previously considered to be a pro-inflammatory prostaglandin, but it may have a much more complex role. PGE_2 _executes its functions by binding mainly to four membrane-bound G-protein-coupled receptors known as E-prostanoid (EP) 1, 2, 3, and 4. These EP receptors have varied effects on cyclic adenosine monophosphate (cAMP) production, phosphoinositol turnover, and intracellular calcium level regulation [1]. The EP1 receptor increases levels of intracellular calcium [2]. The EP3 receptor, which has several isoforms and consequently couples to several different G-proteins, elicits varied signaling pathways that lead to changes in cAMP levels, calcium mobilization, and activation of phospholipase C [3]. The EP2 and EP4 receptors, which increase the intracellular levels of cAMP, work via a G-protein-coupled mechanism that stimulates adenylyl cyclase [4-6].

In mice, the EP2 receptor has been reported to be highly expressed in cerebral cortex, striatum, and hippocampus [7,8]. Genetic knockout of this receptor significantly increases lesion volume at the 24-h time point in mice subjected to ischemic paradigms, with no apparent change in behavior [9,10]. It is important to understand the etiopathology of stroke damage and especially its inflammatory cascade over time. Therefore, our goal was to determine whether the anatomical protective effects of EP2 activation are sustained over time (and are not only transient or delayed) and whether such changes correlate with neurologic improvements.

Furthermore, even though previous studies that used EP2 knockout (EP2^-/-^) mice reported that the presence of EP2 is beneficial in ischemic stroke *in vivo*, none has demonstrated that EP2 stimulation indeed limits infarct damage. To test this paradigm, we investigated whether the highly selective EP2 agonist ONO-AE1-259-01 could reduce ischemic brain damage in C57BL/6 WT mice. ONO-AE1-259-01 has no detectable affinity to any other prostaglandin receptor [11] and binds with higher affinity to the EP2 receptor (Ki = 3 nM) than does PGE_1_, 16,16-dimethyl-PGE_2_, 11-deoxy-PGE_1_, butaprost, or AH-6809 (Ki = 10, 17, 45, 110, and 350 nM, respectively) [5,6,12].

## Materials and methods

### Animals and Treatments

These studies were carried out in male C57BL/6 mice (25 to 30 g) purchased from Charles River Laboratories, Inc (Wilmington, MA). The EP2^-/- ^mouse colony was maintained in the Johns Hopkins animal facility. Animal protocols for these studies were approved by the Johns Hopkins University Animal Care and Use Committee. The animals were allowed free access to water and food before and after surgery. ONO-AE1-259-01 [(16S)-9-deoxy-9beta-chloro-15-deoxy-16-hydroxy-17,17-trimethylene-19,20-didehydro-PGE_2 _sodium salt] was provided by Ono Pharmaceutical Co. Ltd.

### Cerebral Vessel Diameter and Anatomy

To determine the large cerebral vessel gross anatomy in WT and EP2^-/- ^mice, three naïve mice of each genotype were anesthetized deeply and perfused via the heart left ventricle with 5 mL of ice-cold saline followed by 1 mL of black latex paint. Then the mice were decapitated and their brains removed with the circle of Willis intact. The brains were placed in 10% formalin for 24 h before examination with MetaVue software (Meta Imaging Series Software, Downingtown, PA).

### Experimental Design and Drug Injection

In this study, three sets of experiments were performed. In the first experiment, WT (n = 9) and EP2^-/- ^(n = 14) mice were subjected to 90 min of transient middle cerebral artery occlusion (tMCAO) and 96 h of reperfusion. In the second experiment, WT mice (n = 9/group) were given intracerebroventricular injections of ONO-AE1-259-01 (0.5, 1.0, 2.0 nmol) or vehicle (water) 45-50 min before tMCAO. Briefly, mice were anesthetized and mounted on a stereotaxic frame, the skull was exposed under aseptic conditions, and a hole was drilled according to the coordinates: anteroposterior, 0.5 mm; lateral, 1.0 mm from the bregma; and ventral, 2.5 mm relative to the dura. ONO-AE1-259-01 or vehicle was injected in a volume of 0.2 μL into the right lateral ventricle; the needle was left in place for 10 min before being slowly retracted. Finally, the hole was blocked, and the skin overlying the skull was sutured. Mice were then prepared for tMCAO surgery as described below. Physiologic studies were carried out in a separate cohort of correspondingly treated EP2^-/- ^and ONO-AE1-259-01-treated mice. In the third experiment, WT (n = 8) and EP2^-/- ^(n = 7) mice were subjected to distal permanent middle cerebral artery occlusion (pMCAO).

### Transient Focal Cerebral Ischemia (tMCAO) and Reperfusion

Transient focal cerebral ischemia was induced by occlusion of the middle cerebral artery (MCA) with an intraluminal filament, as described previously [13]. Each mouse was maintained with continuous-flow 1.0-1.5% halothane (after induction with 3.0% halothane) in oxygen-enriched air via a nose cone. The core body temperature (rectal) was maintained at 37.0 ± 0.5°C by a heating pad. No differences in rectal temperature between genotypes were noted before, during, or immediately after ischemia. Relative cerebral blood flow (CBF) was measured by laser-Doppler flowmetry (Moor Instruments, Devon, England) with a flexible fiberoptic probe affixed to the skull over the parietal cortex supplied by the MCA (2 mm posterior and 6 mm lateral to the bregma). Under aseptic conditions, the neck and carotid bifurcation were dissected, and the common carotid artery was temporarily ligated. A 7-0 Ethilon nylon monofilament (Ethicon, Inc., Somerville, NJ) coated with flexible silicone (Cutter Sil light universal hardener, Heraeus Kulzer GmbH, Hanau, Germany) was inserted to occlude the MCA. The filament was advanced through an incision in the external carotid artery stump, through the internal carotid artery to the origin of the MCA; successful occlusion was documented by a decrease in laser-Doppler signal of at least 80%. The filament was left in position for 90 min. During occlusion, the neck was closed with sutures, anesthesia was discontinued, and the animals were transferred to a temperature-controlled chamber to maintain the body temperature at 37.0 ± 0.5°C. At 90 min of occlusion, the mouse was briefly re-anesthetized with halothane, and reperfusion was achieved by slowly withdrawing the filament. After its neck was sutured, the mouse was again placed in the temperature-controlled chamber for 2 h and then returned to its home cage for 4 days. The 4-day time point was selected because it allows maximal survival following the tMCAO.

### Permanent Distal Middle Cerebral Artery Occlusion (pMCAO)

Permanent MCAO studies were carried out by the method of Majid *et al. *[14,15] with minor modifications. This permanent distal ischemic protocol was selected because it is highly reproducible and it affects mainly the cortical region, whereas the transient ischemic model causes striatal damage that then extends to the cortical region. The permanent model also allows the study of more distal time points (as described below). Briefly, mice were anesthetized with halothane, and a 1.0-cm vertical skin incision was made between the right eye and ear. The temporal muscle was moved aside to expose the temporal bone. Under a surgical microscope, a 2.0-mm burr hole was drilled over the MCA, transparently visible through the temporal bone. The distal part of the MCA was occluded with a bipolar coagulator, and complete interruption of blood flow at the occlusion site was confirmed by severance of the occlusion site of the MCA. Core body temperature was maintained between 36.5 and 37.5°C during and after the procedure. Animals not circling toward the paretic side after the onset of ischemia and those that developed subarachnoid hemorrhage were eliminated from the study. The successful occlusion was confirmed by placing the laser-Doppler probe above the temporal ridge to establish that blood flow into the region was stopped. At 7 days, mice were euthanized and the brains sectioned. The 7-day time point was selected rather than the 24-h time point because it allows time for extended permanent ischemic damage and can be achieved with 100% survival.

### Assessment of Neurologic Function and Physiologic Parameters

Mice subjected to tMCAO were evaluated for neurologic deficit via a 5-point scale after 96 h of reperfusion. The scores were recorded as: 0, no deficit; 1, forelimb weakness and torso turning to the ipsilateral side when held by the tail; 2, circling to the affected side; 3, unable to bear weight on the affected side; and 4, no spontaneous locomotor activity, according to the protocol used in our previous studies [13,15-17,19]. In a separate cohort, physiologic parameters were measured before occlusion, during occlusion, and during reperfusion. A catheter was inserted into the femoral artery and attached to an automated blood pressure monitor to measure mean arterial blood pressure (MABP). At regular intervals, blood samples were collected through the catheter for analysis of pH, PaO_2_, and PaCO_2_.

Mice subjected to pMCAO were tested for neurologic deficits 7 days after occlusion by an experimenter blinded to the mouse genotype according to a 28-point scoring system [15,20]. Since the pMCAO lesion is distinct from that which occurs with the tMCAO model, we have optimized a neurobehavioral test that enables us to look for subtle and reproducible differences between groups. The tests included assessments of body symmetry, gait, climbing, circling behavior, front limb symmetry, compulsory circling, and whisker response. Each test was graded from 0 to 4, establishing a maximum deficit score of 28. Immediately after the testing, the mice were sacrificed for infarct volume analysis.

### Quantification of Infarct Volumes

After tMCAO and pMCAO, mice were deeply anesthetized, and brains were harvested and cooled in a deep freezer. Five coronal sections of 2-mm thickness were cut and then incubated in 2% 2,3,5-triphenyl-tetrazolium chloride (TTC) in saline for 20 min at 37°C. The area of infarct, which remains white, was measured on the rostral and caudal surfaces of all five slices and numerically integrated across the thickness of the slice to obtain an estimate of infarct volume (SigmaScan Pro, SPSS Inc. Port Richmond, CA). Infarct volume was converted by multiplying the measured infarct volume by the ratio of the contralateral structure to the ipsilateral structure [21].

### Statistics

Data, expressed as mean ± SEM, were analyzed by ANOVA and when appropriate, Newman-Keuls multiple range test. Statistical significance was set at *P *< 0.05.

## Results

### Comparison of Cerebrovascular Anatomy in WT and EP2^-/- ^Mice

**Figure 1:**
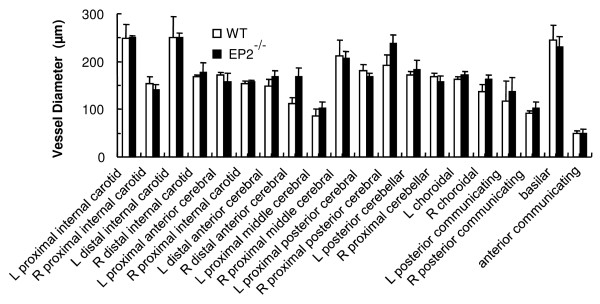
**Genetic deletion of the EP2 receptor does not significantly alter gross vascular anatomy of the brain**. Macroscopic analysis of cerebral arterial vasculature revealed no differences in the circle of Willis or major cerebral arteries between EP2^-/- ^and WT mice (*n *= 3/group).

We evaluated the gross cerebrovascular anatomy by measuring the large vessel diameters in the brains of WT and EP2^-/- ^mice and found no significant differences between the two genotypes (Figure 1). This observation suggested that there are no obvious changes in blood vessel diameters of these mice under this experimental design. This knowledge made us confident to pursue the following ischemic stroke paradigms.

### Effect of EP2 Receptor Genetic Deletion on Monitored Physiologic Parameters of Mice Subjected to tMCAO

**Figure 2:**
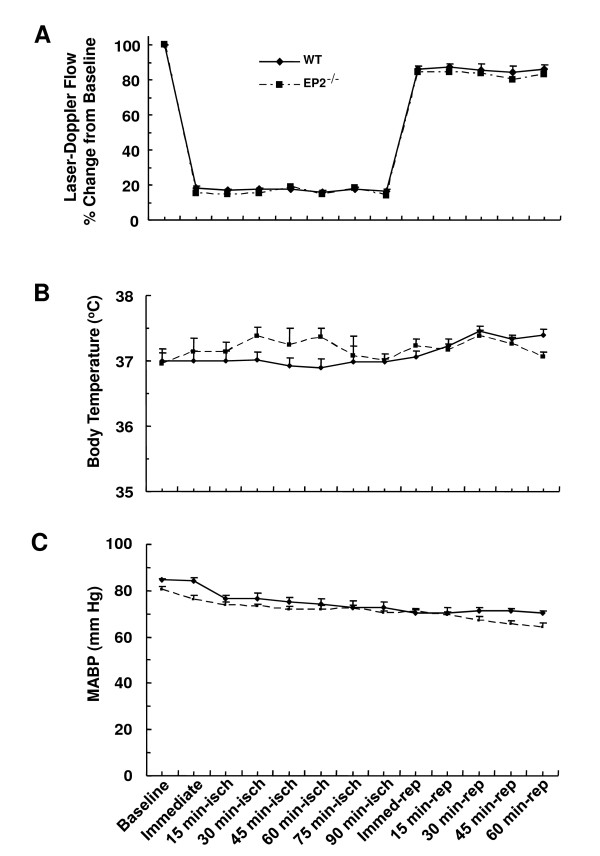
**Genetic deletion of the EP2 receptor does not affect physiologic parameters**. Relative cerebral blood flow (CBF, A), core body temperature (B), and mean arterial blood pressure (MABP, C) were recorded at baseline, at induction of ischemia, and at 15-min intervals during ischemia and 1 h of reperfusion in WT and EP2^-/- ^mice (*n *= 4 WT and 5 EP2^-/-^). The change in CBF was recorded as a percent of baseline.

No significant differences in blood gases (PaO_2_, PaCO_2_, pH) were noted between WT and EP2^-/- ^mice at baseline, 1 h after tMCAO, or 1 h after reperfusion (Table 1). The relative CBF dropped more than 80% from baseline after tMCAO and returned to near baseline after reperfusion in WT and EP2^-/- ^mice (Figure 2). Our data suggest an immediate reperfusion after removal of the filament, whereas a previous report documented a slower recovery that could have potentially affected the stroke outcomes [9]. Overall, no significant differences in CBF, body temperature, or MABP were observed between the two genotypes before, during, or after tMCAO.

**Table 1:** Physiologic Parameters in WT and EP2^-/- ^Mice

Parameter	WT Mice			**EP2**^**-/- **^**Mice**		
	
	Baseline	1 h MCAO	1 h Reperfusion	Baseline	1 h MCAO	1 h Reperfusion
pH	7.37 ± 0.02	7.34 ± 0.02	7.35 ± 0.01	7.32 ± 0.01	7.33 ± 0.01	7.33 ± 0.01
PaCO_2_	38.2 ± 1.4	40.0 ± 1.5	38.7 ± 1.5	42.5 ± 1.3	41.2 ± 2.2	41.4 ± 1.0
PaO_2_	113 ± 6	131 ± 3	121 ± 9	127 ± 3	124 ± 5	120 ± 4

Measurements were made in arterial blood samples obtained via femoral catheter. WT, *n *= 4; EP2^-/-^, *n *= 5.

### Effect of EP2 Receptor Genetic Deletion on Neurologic Scores and Infarct Volume after tMCAO

**Figure 3:**
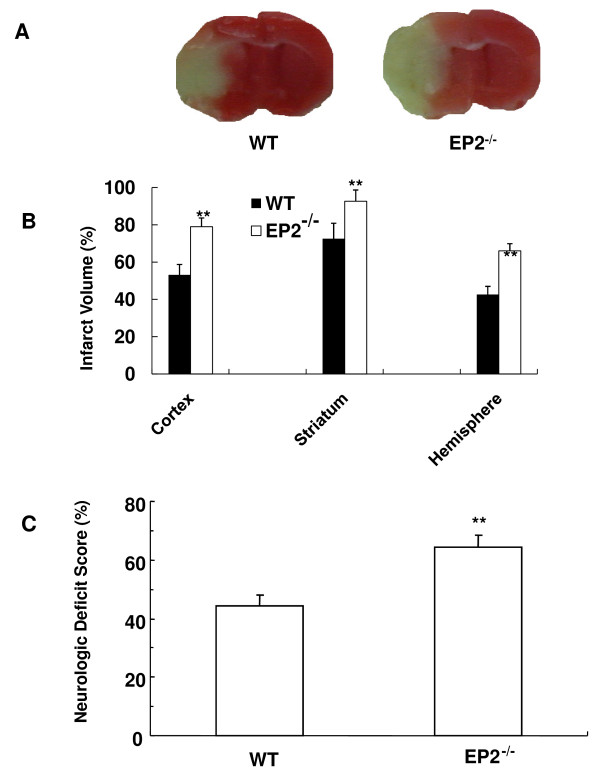
**EP2 receptor deletion increases neurologic deficit scores and infarct volume in mice subjected to tMCAO**. Mice were subjected to 90-min tMCAO and evaluated for neurologic deficits at 4 days. After being scored, the mice were sacrificed, and brain infarction was estimated by TTC staining. (A) Representative photographs show infarcted brain slices from WT (left) and EP2^-/- ^(right) mice. (B) The bar graph shows the corrected cortical, striatal, and hemispheric infarct volumes of WT and EP2^-/- ^mice. The infarct size was significantly larger in EP2^-/- ^(*n *= 14) than in WT mice (*n *= 9). (C) Neurologic deficit scores at 4 days after ischemia were significantly higher in EP2^-/- ^mice than in WT mice, indicating more neurologic dysfunction. **P < 0.01.

The neurologic deficit scores of EP2^-/- ^mice were significantly (*P *< 0.01) higher than those of WT mice after 90-min tMCAO and 4-day reperfusion (Figure 3). Furthermore, according to the TTC-staining method, EP2^-/- ^mice had a significantly larger mean infarct size (*P *< 0.01) than that of their WT counterparts after tMCAO (Figure 3). The percent mortality was estimated at 36% in EP2^-/- ^mice (14 of 22 survived) and 25% in WT mice (9 of 12 survived).

### Effect of the EP2-selective Agonist ONO-AE1-259-01 on Physiologic Parameters of Mice Subjected to tMCAO

The blood gas (pH, PaCO_2_, PaO_2_) concentrations in vehicle- and ONO-AE1-259-01-treated groups (n = 5/group) remained within normal physiologic ranges, and no significant differences were measured between the groups. Similarly, the MABP did not differ significantly between the groups (Table 2). As estimated by laser-Doppler flowmetry, the relative CBF rapidly decreased to more than 80% below baseline in both vehicle- and ONO-AE1-259-01-treated groups. The percent reduction in CBF did not differ significantly between the groups.

**Table 2:** Effect of ONO-AE1-259-01 on Physiologic Parameters

Parameter	Vehicle	0.5 nmol	1 nmol	2 nmol
Pre-ischemia				
pH	7.31 ± 0.02	7.28 ± 0.47	7.30 ± 0.46	7.25 ± 0.23
PaCO_2_	43.2 ± 1.8	44.4 ± 2.0	45.2 ± 1.6	43.0 ± 1.3
PaO_2_	133 ± 5	139 ± 5	130 ± 8	134 ± 9
MABP	78.6 ± 3.9	75.6 ± 1.7	77.4 ± 2.8	72.8 ± 2.5
Ischemia				
pH	7.28 ± 0.02	7.29 ± 0.64	7.26 ± 0.03	7.33 ± 0.02
PaCO_2_	44.0 ± 1.8	43.2 ± 1.5	45.6 ± 2.6	43.16 ± 1.6
PaO_2_	129 ± 5	133 ± 4	124 ± 6	133 ± 5
MABP	74.4 ± 1.7	76.6 ± 2.5	74.4 ± 1.8	74.8 ± 1.7
Reperfusion				
pH	7.26 ± 0.03	7.28 ± 0.05	7.29 ± 0.40	7.28 ± 0.25
PaCO_2_	43.0 ± 1.8	45.8 ± 1.4	47.2 ± 1.3	44.8 ± 2.7
PaO_2_	135 ± 9	129 ± 6	126 ± 7	138 ± 9
MABP	74.6 ± 1.3	73.8 ± 1.7	76.0 ± 1.6	74.0 ± 1.6

Measurements were made in arterial blood samples obtained via femoral catheter (*n *= 5/group).

### ONO-AE1-259-01 Attenuates Neurologic Dysfunction and Infarct Volume in Mice Subjected to tMCAO

**Figure 4:**
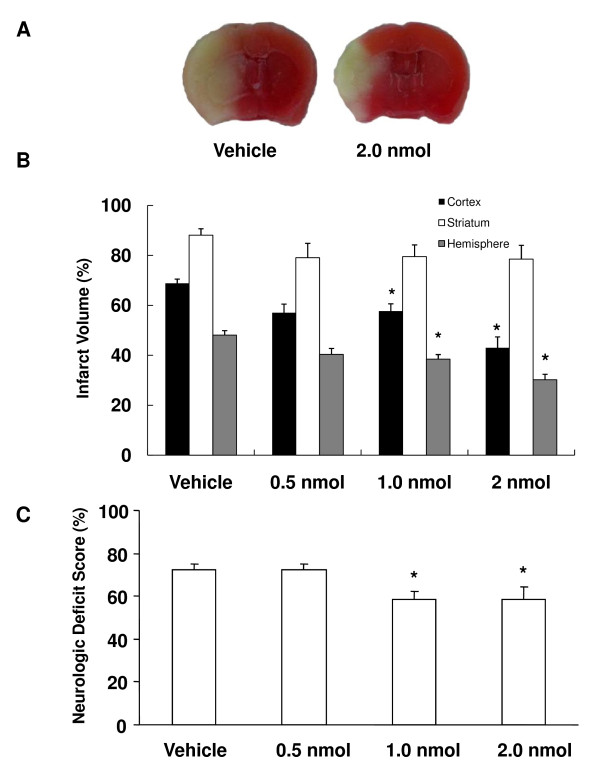
**Effect of pretreatment with ONO-AE1-259-01 on neurologic deficit scores and infarct volume after 4 days of reperfusion**. Mice were pretreated with ONO-AE1-259-01 before being subjected to 90-min MCAO and 4 days of reperfusion. (A) Images of representative TTC-stained brain sections. (B) Percent corrected cortical, striatal, and hemispheric infarct volumes. (C) Neurologic deficit scores were significantly lower in mice treated with 1.0 and 2.0 nmol ONO-AE1-259-01 than in vehicle-treated mice. *n *= 9/group; *P < 0.05 compared with the vehicle-treated group.

Although we and others have shown that deletion of EP2 is detrimental to stroke outcome, here we wanted to test whether the use of a selective agonist would provide protection. The neurologic deficits in the groups administered 1.0 and 2.0 nmol of ONO-AE1-259-01 before tMCAO were significantly less severe than those of the vehicle-treated group; no difference was observed in the group given the lowest dose of 0.5 nmol (Figure 4). Furthermore, the ONO-AE1-259-01-treated groups had significantly (*P *< 0.05) attenuated hemispheric infarct volumes at the 1.0- and 2.0-nmol doses compared with the vehicle-treated group, but the lowest dose did not reach significance (Figure 4). In mice treated with ONO-AE1-259-01, the percent mortality was estimated at 30% at the dose of 0.05 nmol (9 of 13 survived), 30% at 1.0 nmol (9 of 13 survived), and 25% at 2.0 nmol (9 of 12 survived) compared with 36% in the vehicle-treated group (9 of 14 survived).

### Effect of EP2 Receptor Genetic Deletion on Neurologic Deficits and Infarct Volume in Mice Subjected to pMCAO

**Figure 5:**
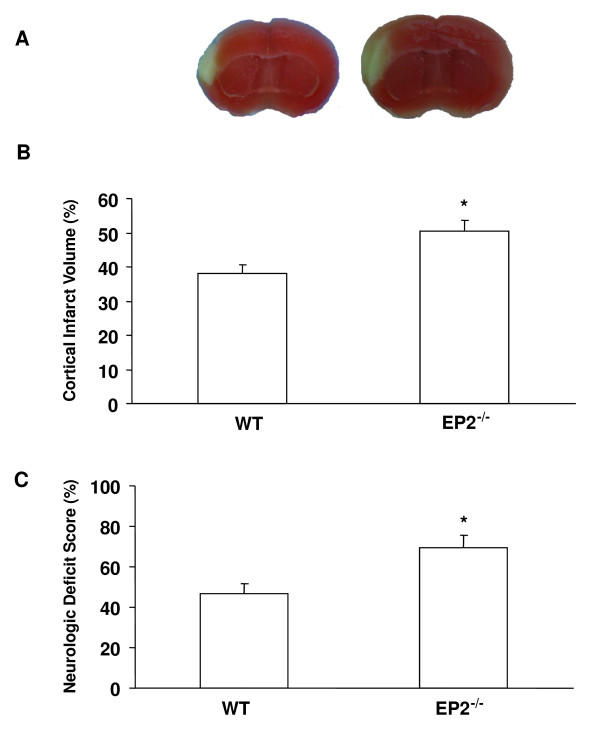
**EP2 receptor deletion increases neurologic deficit scores and infarct volume of mice subjected to pMCAO**. Seven days after being subjected to pMCAO, mice were assessed for neurologic dysfunction and then sacrificed. (A) Representative images show TTC-stained infarcted brain slices from WT (left) and EP2^-/- ^(right) mice. (B) The bar graph shows the corrected cortical infarct volumes of WT and EP2^-/- ^mice. (C) Neurologic deficit scores at 7 days after ischemia were significantly higher in EP2^-/- ^mice (*n *= 7) than in WT mice (*n *= 8). **P < 0.008.

To address whether ischemic outcomes could differ in different ischemic stroke models, we compared outcomes obtained from the transient ischemic reperfusion model (as represented in Figure 3) with those of the permanent ischemic model. The transient model is characterized by an ischemic-reperfusion injury that results consistently in damage that begins mostly in the striatum and then spreads especially to the surrounding cortical region. In contrast, the permanent distal occlusion model does not have the reperfusion injury component, and it results in a brain lesion mainly constrained within the cortex. Thus, these two models are complementary. In addition to addressing different ischemic stroke paradigms, their outcomes are also regionally dependent. Finally, whereas others have studied early time points after stroke, namely 24 h [10], we assessed outcomes at 7 days after pMCAO. We found that at 7 days after pMCAO, EP2^-/- ^mice suffered significantly greater neurologic deficits than did WT mice (*P *< 0.04) and had significantly larger infarct volumes than did their WT counterparts (*P *< 0.008; Figure 5). No mortality was observed after distal permanent focal cerebral ischemia in either genotype.

## Discussion

This study was designed to further ascertain the unique neuroprotective properties of the PGE_2 _EP2 receptor in ischemic stroke. Using EP2^-/- ^mice, we showed that the EP2 receptor is protective in cortical and striatal brain regions affected by tMCAO and in the cortex after pMCAO but that its deletion does not affect the gross cerebrovascular anatomy or the physiologic parameters of blood pH, PaO_2_, PaCO_2_, or MABP. We also demonstrated that this protective action of EP2 receptors is not simply transient or acute, as previously suggested, but lasts for days. Furthermore, pretreatment of mice with 1.0- and 2.0-nmol doses of the highly selective EP2 receptor agonist ONO-AE1-259-01 significantly reduced the infarct volume induced by transient ischemia, again without affecting the physiologic parameters monitored. These results clearly demonstrate that pharmacologic stimulation of the EP2 receptors in the brain attenuates brain damage caused by cerebral ischemia and support the hypothesis that EP2 receptor stimulation is important throughout an extended duration of the pathophysiologic response to brain ischemic damage. Measurements of relative CBF showed that tMCAO caused a reduction in cortical perfusion throughout the ischemic period that was similar in the EP2^-/- ^and drug-treated mice to that in their corresponding controls, suggesting that neither genetic deletion nor pharmacologic activation of the EP2 receptor affected the severity of the ischemic insult. Thus, our findings indicate a protective role for EP2 by mechanisms that are likely other than those involving cerebrovascular effects. Moreover, no significant differences were observed in body temperature or MABP.

EP2^-/- ^mice that underwent tMCAO or pMCAO had greater neurologic disability and infarct size than did WT mice. Although compensatory pathways might occur in the knockout animals, we show here that WT mice that received the two higher doses of the EP2 agonist ONO-AE1-259-01 before tMCAO had significantly less severe neurologic deficits and less infarct damage, supporting observations that EP2 receptors are beneficial in excitotoxicity [8] and in ischemic stroke [9].

Previous reports have shown that the EP2 receptor elicits neuroprotective effects under conditions of excitotoxicity and oxygen-glucose deprivation by increasing intracellular levels of cAMP and activating PKA signaling [9,10]. We have also previously reported that pharmacologic activation of EP2 receptors leads to neuroprotection via the cAMP-PKA pathway [22]. Moreover, the pharmacologic stimulation of EP4/EP3 receptors affords protection by increasing intracellular levels of cAMP and through activation of the ERK pathway [13]. These cAMP cascades can provide protection for example by: (1) reducing the release of endoplasmic Ca^2+ ^through the inositol triphosphate receptor [23], (2) inhibiting the expression of adhesion molecules [24], (3) suppressing the activity of neuronal nitric oxide synthase [25], (4) stimulating cAMP response element binding (CREB) [26], and (5) stimulating the high-affinity glutamate transporter [27]. Interestingly, ONO-AE1-259-01 has been suggested to elevate cAMP and inhibit expression of inflammatory molecules such as ICAM-1 and B7.2 (CD86) [28,29], and such inflammatory molecules affect stroke outcomes [30,31]. The exact cascade *in vivo *is likely to be much more elaborate than what we can begin to address in isolated neuronal cultures, especially considering the complexity of the different cells and interactions between them and their environment (blood flow, oxygenation, inflammation, etc) over time. The signaling pathways of these molecules and kinases are continually expanding (kinome-phosphorylome projects [32]), with many branches that link to other pathways. Therefore, the complete cascade of events that takes place within any given cell based on its location with respect to the infarct is likely to differ substantially. Indeed, it is highly probable that the neuroprotection provided by EP2 stimulation results from a combination of pathways rather than a single one. For these reasons, we have first focused here on demonstrating that in the brain, stimulating the EP2 receptors leads to anatomical and behavioral protection. To build on our work with the tMCAO model, we are now endeavoring to confirm that the EP2-selective agonist is also protective in the pMCAO model and to address potential targets/biosystems that could begin to explain some of the steps leading to neuroprotection.

Two previous studies have indicated that activation of the PGE_2 _EP2 receptor can protect against excitotoxic and anoxic injury [9,10]. In one of those studies, the tMCAO was followed by only 22.5 h of reperfusion, and no behavioral outcomes were reported. It is important to document that such ischemic-reperfusion-related change in the knockout animal is not transient or delayed, and that it is indeed maintained at later time points. When a brief ischemic event is followed by reperfusion, a second phase of injury occurs (potentially mediated by a surge in inflammatory markers). What's more, over time, endogenous repair pathways can be activated. Although it is more challenging to keep a mouse alive for 96 h after transient ischemia, we selected this extended reperfusion time because it enables us to document that the protective role of EP2 is sustained during the entire period. In the other study, Liu *et al. *first tested the stroke outcomes (without behavioral outcomes) in mice subjected to pMCAO after only 24 h of survival [10] and then a subsequent study suggested that misoprostol (which is a poorly selective mouse EP2 receptor agonist) has a protective effect against MCAO injury [33]. For reasons similar to those described above, it is important to determine the potential contributions of EP2 to neurobehavioral and ischemic outcomes at later time points; that is why in our study we selected 7 days. Both of these previous studies led to the suggestion that activation of the PGE_2 _EP2 receptor can protect against ischemic injury, although no data were provided to support this hypothesis. To make such a conclusion, one needs to test whether selectively targeting EP2 would result in neuroprotection. Furthermore, it has been suggested that compensatory mechanisms can occur in knockout animals. Therefore, we chose to directly activate the EP2 receptor with a selective agonist to complement the findings in knockout mice.

Based on these novel observations, we can conclude that pharmacologic stimulation of the EP2 receptor with a selective pharmacologic agent could potentially be used therapeutically in translational medicine (most likely in combination with other standard treatments) to limit brain damage following ischemic stroke.

## Competing interests

The authors declare that they have no competing interests.

## Authors' contributions

SD conceived, designed and coordinated the study; MA and SS participated in the design, performed the experiments and analyzed the data; ZA assisted with the preclinical model; TM and SN contributed the animals, the drugs and reviewed the paper. All authors read and approved the manuscript.
